# Disease-Associated Mutant Ubiquitin Causes Proteasomal Impairment and Enhances the Toxicity of Protein Aggregates

**DOI:** 10.1371/journal.pgen.1000382

**Published:** 2009-02-13

**Authors:** Elizabeth M. H. Tank, Heather L. True

**Affiliations:** Department of Cell Biology and Physiology, Washington University School of Medicine, St. Louis, Missouri, United States of America; Fred Hutchinson Cancer Research Center, United States of America

## Abstract

Protein homeostasis is critical for cellular survival and its dysregulation has been implicated in Alzheimer's disease (AD) and other neurodegenerative disorders. Despite the growing appreciation of the pathogenic mechanisms involved in familial forms of AD, much less is known about the sporadic cases. Aggregates found in both familial and sporadic AD often include proteins other than those typically associated with the disease. One such protein is a mutant form of ubiquitin, UBB+1, a frameshift product generated by molecular misreading of a wild-type ubiquitin gene. UBB+1 has been associated with multiple disorders. UBB+1 cannot function as a ubiquitin molecule, and it is itself a substrate for degradation by the ubiquitin/proteasome system (UPS). Accumulation of UBB+1 impairs the proteasome system and enhances toxic protein aggregation, ultimately resulting in cell death. Here, we describe a novel model system to investigate how UBB+1 impairs UPS function and whether it plays a causal role in protein aggregation. We expressed a protein analogous to UBB+1 in yeast (Ub^ext^) and demonstrated that it caused UPS impairment. Blocking ubiquitination of Ub^ext^ or weakening its interactions with other ubiquitin-processing proteins reduced the UPS impairment. Expression of Ub^ext^ altered the conjugation of wild-type ubiquitin to a UPS substrate. The expression of Ub^ext^ markedly enhanced cellular susceptibility to toxic protein aggregates but, surprisingly, did not induce or alter nontoxic protein aggregates in yeast. Taken together, these results suggest that Ub^ext^ interacts with more than one protein to elicit impairment of the UPS and affect protein aggregate toxicity. Furthermore, we suggest a model whereby chronic UPS impairment could inflict deleterious consequences on proper protein aggregate sequestration.

## Author Summary

The accumulation of cytotoxic protein aggregates occurs in many neurodegenerative diseases. It is difficult to determine if the protein aggregates found in these diseases represent a cause or consequence of the disorder. Degradation pathways, such as the ubiquitin/proteasome system (UPS), remove misfolded proteins that are prone to aggregate. The UPS involves many players that work in concert to target proteins for degradation by the proteasome. A mutant form of ubiquitin has been associated with many diseases, including Alzheimer's disease. We developed a yeast model of the mutant ubiquitin protein in order to investigate its effect on UPS function and protein aggregation. We demonstrate that this mutant ubiquitin causes impairment of the UPS and suggest that it does so by interacting with multiple components of the pathway. Using this model, we evaluated the effects of the mutant ubiquitin on nontoxic protein aggregates and found that they were unaltered by its presence. We demonstrate that the mutant ubiquitin acts as a modifier, which increases cellular susceptibility to the phenotypic effects of deleterious protein aggregates by altering UPS functionality and substrate ubiquitination. Furthermore, the system we developed can be utilized to further understand the complex interplay of proteasomal impairment and protein aggregate toxicity.

## Introduction

As technology and medicine further extend the human lifespan, age-related diseases will become more prevalent. Alzheimer's disease (AD) is a neurodegenerative disorder that affects 20 million people worldwide and is the most common form of late-onset dementia [1]. The study of genetic mutations that cause early onset AD has provided insight into some of the factors involved, but most cases of AD are sporadic and of unknown origin. Uncovering the risk factors involved in any multi-factorial disease is challenging but vital for disease treatment and prevention. Many fundamental pathways, including the ubiquitin proteasome system (UPS), have been suggested to play a role in AD. Therefore, investigating the relationship between AD and the UPS could lead to new therapeutic targets.

The UPS is an evolutionarily conserved pathway that selectively eliminates short-lived and damaged proteins. A number of cellular processes, including the cell cycle, stress response, and DNA repair, require the UPS [2]. Protein degradation by the UPS involves a series of enzymes that ultimately attach ubiquitin, a small well-conserved protein, to an internal lysine residue in the target protein [3]–[5]. Multiple ubiquitin proteins can be connected to form a polyubiquitin chain which serves as a degradation signal recognized by the 26S proteasome. A series of events involving E1, E2 and E3 enzymes are required to attach ubiquitin via its C-terminal glycine residue to the target protein. The formation of polyubiquitin chains and the process of ubiquitin conjugation to protein targets displays exquisite specificity, in part by the multitude of E2 and E3 enzymes. Despite intensive study, the roles of many components of the UPS remain to be elucidated.

The importance of the UPS in cellular homeostasis is apparent not only by the redundancy and conservation of the components, but also by its role in disease [5],[6]. The complex interplay between protein aggregation and UPS function is easily appreciated, yet it is often difficult to determine the causal nature of the problem. UPS dysfunction can prevent the degradation of misfolded proteins, which can lead to aggregation. Conversely, protein aggregates can be challenging substrates for the UPS and can thus cause proteasomal impairment [7]. Protein aggregation is a hallmark of many neurodegenerative disorders [6]. In addition, mutations in ubiquitin processing enzymes, such as UCHL1 and Parkin, can lead to inherited forms of neurodegenerative diseases [8],[9]. Furthermore, many protein aggregates associated with disease show ubiquitin deposition [10], suggesting that dysfunctional UPS activity may contribute to pathogenesis. Understanding the interplay between protein aggregation and clearance is an active area of research, but most systems are complicated by cellular toxicity, which alone can have negative consequences on protein homeostasis.

A mutant form of ubiquitin was found associated with AD and other diseases and was proposed to act as a natural proteasome inhibitor [11]. The generation of this mutant ubiquitin protein is unusual - the mutation is found in the messenger RNA, but not in the DNA sequence of the ubiquitin-B gene. The mutant ubiquitin results from a dinucleotide deletion near the 3′ end of the mRNA transcript which shifts the reading frame for translation. The mutant protein has been named UBB+1 [12]. The dinucleotide deletion event in the mRNA has been termed “molecular misreading”, though the mechanism by which the deletion occurs remains elusive [13],[14]. Many human mRNA transcripts, including all copies of ubiquitin, contain potential sites for molecular misreading, since hotspots for these events are hypothesized to occur near simple repeat sequences (e.g. GAGAG) [15]. The best characterized +1 mutant ubiquitin protein has a short C-terminal extension, with the majority of the protein being identical to ubiquitin [12]. As such, the protein is presumably folded and recognized as ubiquitin, but the C-terminal glycine residue essential for conjugation to substrates is absent.

The accumulation of the UBB+1 protein in the neurological hallmarks of AD is curious, since the mutant cannot be conjugated to target proteins [12]. The presence of UBB+1 has been proposed to represent an endogenous readout of proteasomal dysfunction [16],[17]. Due to its association with protein aggregation, it was also suggested that UBB+1 could contribute to disease pathology [18]. UBB+1 protein accumulation has been documented in multiple disorders such as polyglutamine expansion diseases (including Huntington's disease), Pick's disease and even non-neuronal tissue diseases [11],[19]. However, the mechanism of UBB+1 action in these diseases remains unclear.

To evaluate the role of UBB+1 in disease, the effects of ectopic UBB+1 expression have been investigated in cultured mammalian cells. Although UBB+1 cannot be conjugated to target substrates, it can be ubiquitinated by wild type ubiquitin and degraded by the proteasome [20]. However, high levels of UBB+1 expression cause proteasomal impairment [16],[21],[22]. As a natural inhibitor of the UPS, UBB+1 could be another example whereby proteasomal impairment induces protein aggregation. Therefore, UBB+1 might act as a disease modifier. Recently, a UBB+1 transgenic mouse has been characterized [23]. UBB+1 expression resulted in constant UPS impairment that caused a minor learning deficit and caused changes in transcription profiles that mirror those found in brains of humans with AD [23]. The expression of UBB+1 in mammalian cells enhances the toxicity and aggregation of an expanded polyglutamine protein [24]. However, measuring changes in protein aggregation in cells that are dying from toxic protein aggregates is challenging. Hence, it remains to be determined if UBB+1 affects protein aggregation *per se*, or if it affects the ability of the cells to cope with the aggregates.

We developed a model system using *Saccharomyces cerevisiae* to evaluate the cellular effects of UBB+1. We expressed a mutant ubiquitin protein (Ub^ext^) analogous to UBB+1 and found that it caused UPS impairment in yeast. Furthermore, we found that Ub^ext^ changed the ubiquitination pattern on a UPS substrate. Taking advantage of non-toxic protein aggregates in yeast, we demonstrated that the expression of Ub^ext^ neither induced nor changed these aggregates. However, Ub^ext^ did make cells more susceptible to toxic protein aggregates. We propose that Ub^ext^ does not cause protein aggregation, but rather acts as a phenotypic enhancer of deleterious aggregation. We present a model based on our work and other recent advances in the field to explain how this might occur.

## Results

### Ub^ext^ Expression in Yeast Cannot Functionally Rescue a Decrease in Wild Type Ubiquitin

**Figure 1:**
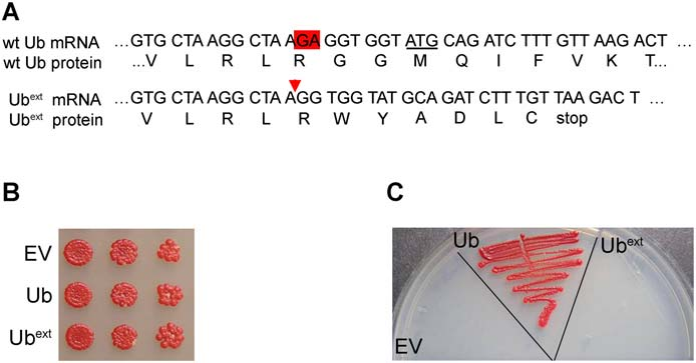
Ub^ext^ does not function as ubiquitin. (A) A schematic diagram depicting the wild type and mutant ubiquitin (Ub^ext^) mRNA and protein sequences beginning at nucleotide 207 of *UBI4*. The underlined ATG denotes the beginning of the next ubiquitin open reading frame in the tandem array. The red triangle signifies the site of the dinucleotide GA deletion. (B) Ub^ext^ expression does not affect logarithmically growing yeast. Serial dilutions of wild type yeast ectopically expressing Ub^ext^, excess wild type ubiquitin (Ub), or an empty vector (EV) control were spotted onto selective medium. (C) Ub^ext^ does not behave as wild type ubiquitin and cannot compensate for the loss of *UBI4*. The *Δubi4* strain was transformed with EV, Ub, and Ub^ext^. Transformants were plated onto selective medium following growth into stationary phase.

The mechanism by which +1 proteins, such as UBB+1, are produced is currently unknown. To create a yeast model of UBB+1, we generated an expression vector containing the sequence of the first ubiquitin-coding region of the yeast tandem ubiquitin gene, *UBI4*, such that a dinucleotide deletion occurred near the carboxy terminus (Figure 1A). The deletion caused a frameshift in the coding sequence of ubiquitin and extended the open reading frame to the next stop codon (termed extended ubiquitin or Ub^ext^). This construct mimics the generation of UBB+1 from the human tandem ubiquitin gene (ubiquitin-B). Constitutive expression of Ub^ext^ in log-phase yeast did not cause a growth defect when assessed in either liquid medium (data not shown) or on solid medium (Figure 1B). Wild type cells expressing Ub^ext^ did show a reduced growth rate after recovery from stationary phase (data not shown).

To evaluate the functionality of Ub^ext^, we analyzed its ability to replace wild type ubiquitin. The stress-inducible *UBI4* gene encodes a tandem array of five ubiquitin moieties that are separated post-translationally by deubiquitinating enzymes (DUBs) that cleave after the C-terminal glycine residue, G76 [25]. *UBI4* is non-essential in vegetatively growing cells but is required for cells to recover from various stress conditions [26],[27]. We utilized a strain lacking *UBI4* to evaluate the functionality of Ub^ext^. Δ*ubi4* cells were transformed with expression plasmids that contain wild type ubiquitin, Ub^ext^ or empty vector. The transformants were grown for two weeks to allow them to reach stationary phase and then plated again to evaluate their ability to recover. Only cells expressing extra wild type ubiquitin were rescued from the loss of *UBI4* and could grow after this stress (Figure 1C). This demonstrates that Ub^ext^ is a non-functional ubiquitin, as expected due to the lack of the C-terminal glycine residue required for conjugation to target substrates.

### Ub^ext^ Expression Causes UPS Impairment

**Figure 2:**
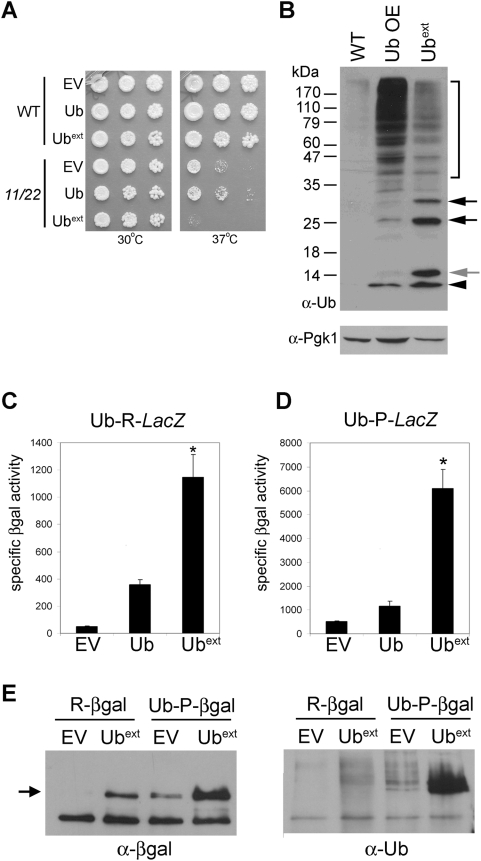
Expression of Ub^ext^ causes proteasomal impairment. (A) Ub^ext^ displays synthetic lethality with proteasome mutants. Wild type (WT) and temperature-sensitive proteasome mutant cells, *pre1-1 pre2-2* (11/22), were transformed with plasmids containing empty vector (EV), ubiquitin (Ub) and Ub^ext^. Serial dilutions of cells were spotted onto selective medium and grown at 30°C and 37°C. (B) Cells expressing Ub^ext^ show a distinct pattern of ubiquitin conjugation. Protein lysate from wild type yeast cells containing an empty vector (WT), extra ubiquitin (Ub OE), or Ub^ext^ were analyzed by SDS-PAGE and western blot using an anti-ubiquitin antibody. Ub^ext^ causes an increase in ubiquitin-conjugated proteins (bracket) as compared to WT. The black arrowhead indicates ubiquitin monomer. The grey arrow points to Ub^ext^. Black arrows represent conjugated Ub^ext^. Pgk1p expression was probed to assess protein loading on the membrane. (C) Ub^ext^-expressing cells impair the degradation of the N-end rule substrate R-β-galactosidase (βgal). Cells containing EV, Ub, or Ub^ext^ were transformed with pGal-Ub-R-*LacZ*. The stability of R-βgal was measured by specific activity (luminescence units/µg protein). The asterisk (*) indicates statistical significance between wild type Ub and Ub^ext^ (p = 0.0013). (D) Ub^ext^-expression prevents the efficient proteasomal degradation of a ubiquitin fusion degradation substrate. The stability of Ub-P-*LacZ* was evaluated as in B. The asterisk (*) indicates statistical significance between wild type Ub and Ub^ext^ (p = 0.0005). (E) Ubiquitinated reporter substrates are present in Ub^ext^-expressing cells. Wild type cells containing the Ub-X-*LacZ* reporter constructs and expressing Ub^ext^ or the control (EV) were analyzed for ubiquitinated βgal protein. βgal protein was immunoprecipitated with an anti-βgal antibody (left) and the bound fractions were blotted with an anti-ubiquitin antibody (right). The arrow indicates full length βgal protein.

If Ub^ext^ affects UPS functionality in yeast as UBB+1 does in mammals, then we hypothesized that Ub^ext^ would display synthetic lethality with a proteasome mutant. We evaluated the cellular viability of a temperature-sensitive catalytic proteasome mutant strain (*pre1-1 pre2-2*) [28] expressing Ub^ext^. As predicted, Ub^ext^-expressing *pre1-1 pre2-2* cells were inviable at the restrictive temperature (Figure 2A). Wild type cells expressing Ub^ext^ grown at the restrictive temperature did not show a growth defect (Figure 2A). Next we evaluated another ubiquitination-dependent process to determine if Ub^ext^ effects are more widespread. We challenged Ub^ext^-expressing cells to DNA damage induced by UV irradiation and found that they survived as well as the control cells (data not shown).

Ub^ext^ cannot be conjugated to target protein substrates, but can be recognized as a UPS substrate. Therefore, we assessed its ubiquitination. Protein lysate from Ub^ext^-expressing cells and control cells were evaluated by SDS-PAGE and western blot. Cells expressing Ub^ext^ exhibited a unique band which represents the extended mutant ubiquitin protein (Figure 2B, grey arrow) which is larger than wild type ubiquitin (Figure 2B, arrowhead). Cells expressing Ub^ext^ also displayed a distinctive laddering pattern which suggests that Ub^ext^ is conjugated by wild type ubiquitin moieties (Figure 2B, black arrows). A similar laddering pattern was previously observed in cells expressing UbΔGG [29], a mutant ubiquitin protein lacking only the two C-terminal glycine residues, and we observed the same pattern when we expressed UbΔGG in yeast (data not shown). Additionally, a strain lacking the ubiquitin recycling DUB (Δ*ubp14*) accumulates free ubiquitin chains [29] and we also observed that Δ*ubp14* cells show the same ubiquitin laddering pattern as cells expressing Ub^ext^ (data not shown).

The expression of Ub^ext^ also caused an increase in the level of unconjugated wild type ubiquitin, which was evident by the accumulation of the mono-ubiquitin band in the Ub^ext^ lane in comparison to the empty vector control lane (Figure 2B, black arrowhead). Further analysis by quantitative western blot showed approximately a 10-fold increase in wild type mono-ubiquitin in the presence of Ub^ext^ (data not shown). Transcriptional activity from the *UBI4* promoter using a *UBI4promoter-LacZ* reporter in Ub^ext^-expressing cells demonstrated a modest two-fold increase (data not shown), suggesting that *UBI4*-induced transcription may be one, but perhaps not the only source for the increased ubiquitin. Cells expressing Ub^ext^ also displayed an increase in the abundance of high molecular weight ubiquitin-conjugated proteins in comparison to the empty vector control (Figure 2B, compare left lane WT to right lane Ub^ext^). The fact that Ub^ext^ caused lethality in the proteasome mutant strain and Ub^ext^-expressing cells accumulated ubiquitinated-protein conjugates, suggests that it is affecting protein degradation. An accumulation of high molecular weight ubiquitinated proteins also occurred with the over expression of wild type ubiquitin (Figure 2B, middle lane). Most likely this occurs because of more ubiquitination of endogenous proteins due to an excess of functional ubiquitin provided by the over expression construct.

We tested the functionality of the UPS in cells expressing Ub^ext^ using two different proteasome reporters constructs: an N-end rule substrate and a ubiquitin fusion degradation (UFD) substrate [30]. These substrates are processed by the UPS using distinct enzymes [3],[31],[32]. The N-end rule substrate is a Ub-R-*LacZ* fusion. The ubiquitin moiety is efficiently cleaved by endogenous DUBs to expose the N-terminal amino acid (arginine) of β-galactosidase (βgal). According to the N-end rule, R-βgal is an unstable protein that is polyubiquitinated and rapidly degraded by the 26S proteasome [33]. The UFD reporter substrate is Ub-P-*LacZ*. In yeast, no DUB can cleave ubiquitin from βgal if the first amino acid after ubiquitin is proline. Because of the ubiquitin fusion, Ub-P-βgal is unstable and is rapidly degraded by the proteasome. These constructs, along with a stable *LacZ* control (Ub-M-*LacZ*), were transformed into cells expressing Ub^ext^ to assess UPS function by βgal activity assays. Cells expressing Ub^ext^ and either of the unstable proteasome reporters displayed higher levels of specific βgal activity (Figure 2C and 2D). Cells expressing extra wild type ubiquitin showed a slight increase in the stabilization of the reporter constructs. The expression of extra wild type ubiquitin also generated a large steady state population of ubiquitin-conjugated proteins (Figure 2B, middle lane), which could be taxing the degradation capacity of the proteasome. To evaluate if *LacZ* fusion expression was affected by Ub^ext^, stable M-βgal activity was measured and showed no difference (data not shown). These results demonstrate that the expression of Ub^ext^ in yeast inhibits the degradation of two different UPS reporter substrates.

Such stabilization of the proteasome reporter constructs could be due to a lack of ubiquitination of the reporter, since the expression of Ub^ext^ also causes accumulation of unconjugated wild type ubiquitin. The reporter substrates (βgal protein) were immunoprecipitated from cells with and without the co-expression of Ub^ext^. Western blot with an anti-βgal antibody revealed that more β-gal protein was precipitated in Ub^ext^-expressing cells (Figure 2E, left). This result correlates with the higher levels of βgal activity measured in Ub^ext^-expressing cells (Figure 2C and D). Analysis with an anti-ubiquitin antibody showed ubiquitin-conjugated R-βgal and Ub-P-βgal in cells expressing Ub^ext^ (Figure 2E, right). This data demonstrates that Ub^ext^ is not stabilizing these UPS substrates by blocking their ubiquitination.

### Expression of Ub^ext^ Does Not Directly Block Proteasome Function

**Figure 3:**
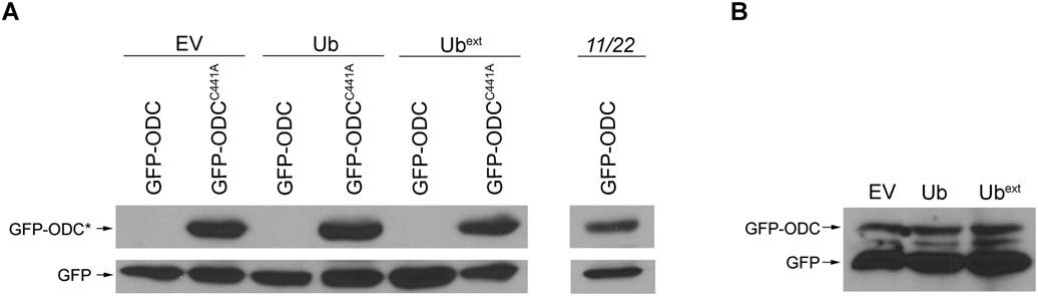
Ub^ext^-expressing cells can degrade a ubiquitin-independent substrate. (A) Expression of Ub^ext^ does not impair the degradation of GFP-ODC. Cells containing a stable GFP construct were transformed with empty vector (EV), Ub, or Ub^ext^. These cells were then transformed with plasmids expressing either GFP-ODC or GFP-ODC^C441A^. A proteasome mutant strain (*pre1-1 pre2-2*, abbreviated *11/22*) was transformed with both the GFP and GFP-ODC constructs and as expected, both were stable. Protein lysates were separated by SDS-PAGE and analyzed by western blot using an anti-GFP antibody. GFP-ODC* denotes either GFP-ODC or GFP-ODC^C441A^. (B) Ub^ext^-expressing cells accumulate approximately an equal amount GFP-ODC in comparison to controls. Protein lysates from cells containing EV, Ub or Ub^ext^ co-expressing GFP-ODC were analyzed by SDS-PAGE and western blot with an anti-GFP antibody and visualized after prolonged exposure (1 hour).

Another plausible explanation for the UPS inhibition could be that Ub^ext^ binds to the proteasome and this interaction precludes other proteasome substrates from being efficiently degraded. Alternatively, Ub^ext^ could interact with other component(s) of the UPS and inhibit their function. To examine whether Ub^ext^ is clogging the proteasome, we took advantage of a ubiquitin-independent proteasome substrate. Ornithine decarboxylase (ODC) is an enzyme involved in polyamine biosynthesis [34],[35] and a short peptide from this protein serves as a ubiquitin-independent degradation signal (i.e. degron) [36]. Measuring the degradation of ODC reflects the functionality of the proteasome in a manner independent of the non-proteasomal components of the UPS cascade. A fusion of GFP with the degron of ODC (GFP-ODC) serves to target GFP to the proteasome where it is rapidly degraded [37]. A point mutation in the ODC degron (C441A) stabilizes the fusion protein by lowering its affinity for the proteasome [38],[39]. GFP-ODC fusions were transformed into cells expressing Ub^ext^ and the steady state level of GFP-ODC was evaluated by western blot (Figure 3A). Cells expressing Ub^ext^ were able to degrade the GFP-ODC protein while the stable GFP-ODC^C441A^ protein accumulated (Figure 3A). Even prolonged exposure showed that the steady state level of GFP-ODC was approximately equal with or without Ub^ext^ expression (Figure 3B). Thus, Ub^ext^ permits the degradation of a ubiquitin-independent proteasome substrate, suggesting that the proteasomal degradation capacity is not significantly impaired in cells expressing Ub^ext^.

### Simple Modifications Do Not Alleviate the UPS Impairment Caused by Ub^ext^

We sought to determine how Ub^ext^ exerts its negative effects on the UPS pathway. We asked whether Ub^ext^ was sequestrating wild type ubiquitin proteins. Ubiquitinated-Ub^ext^ could be refractory to DUBs, thereby tying up ubiquitin, as suggested for UBB+1 [20]. To test this hypothesis, we expressed extra ubiquitin in the presence of Ub^ext^ and found that the UPS test substrates were still stabilized (data not shown). This result was not surprising since monomeric ubiquitin appears to be abundant in cells expressing Ub^ext^ (Figure 2B, arrowhead). This suggests that a lack of wild type ubiquitin is not the cause of the UPS impairment elicited by Ub^ext^.

Ub^ext^ lacks the essential C-terminal glycine residues (G75 and G76) required for ubiquitin conjugation and these glycine residues are vital for many proteins to interact with ubiquitin [40]. We tested whether adding back two glycine residues to the C-terminal extension of Ub^ext^ (Ub^ext^+GG) could restore these interactions and alleviate the proteasomal impairment. Cells expressing Ub^ext^+GG still displayed proteasomal impairment (data not shown), indicating that the C-terminal extension plays a mechanistic role in the phenotype observed.

### Ub^ext^-Ubiquitin Conjugation Is Required for N-End Rule Substrate Stabilization but not for UFD Substrate Stabilization

**Figure 4:**
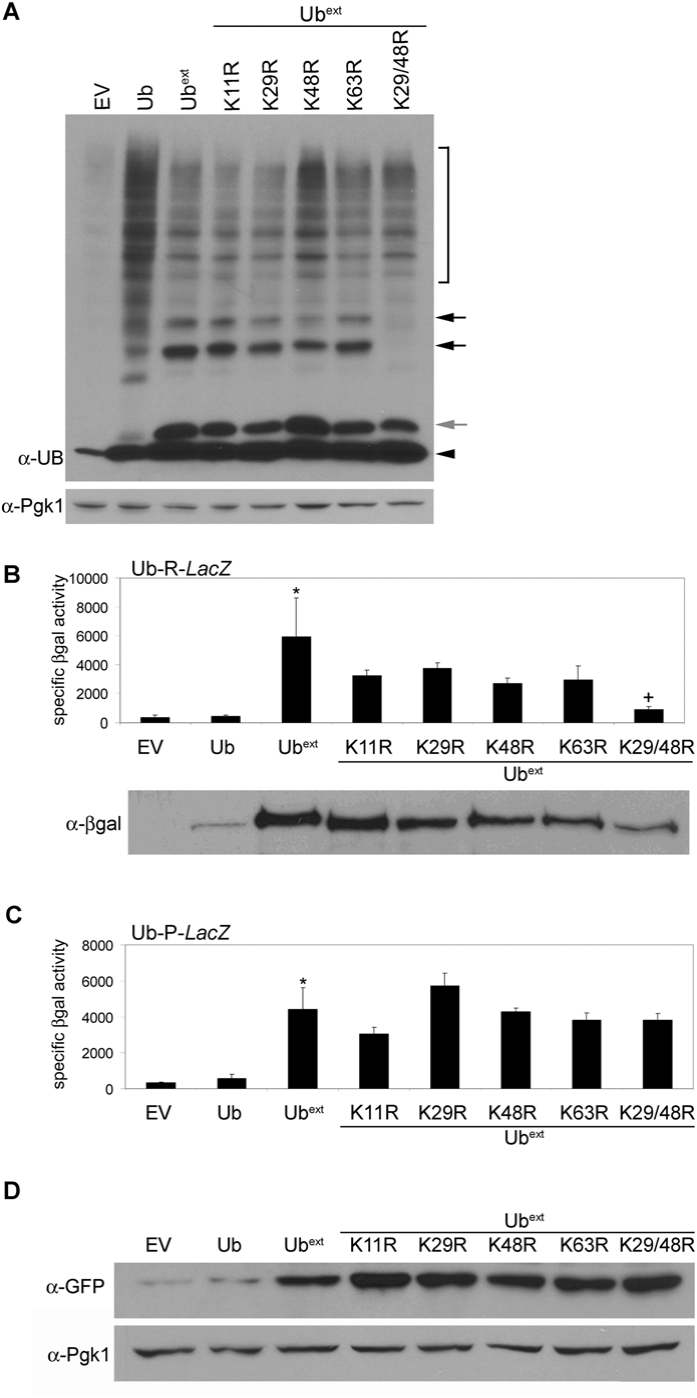
Ubiquitin conjugation of Ub^ext^ is required for stabilization of N-end rule but not UFD substrates. (A) Lysines 29 and 48 are required for ubiquitin conjugation to Ub^ext^. Protein lysate from cells containing empty vector (EV), Ub, Ub^ext^, or Ub^ext^KxR were analyzed by SDS-PAGE and western blot using an anti-ubiquitin antibody. The black arrowhead indicates mono-ubiquitin. The grey arrow points to Ub^ext^. Black arrows represent conjugated Ub^ext^. Pgk1p was probed to assess protein loading (lower). (B) The conjugation of Ub^ext^ is necessary for the impaired degradation of the N-end rule substrate R-βgal. Cells containing EV, Ub, Ub^ext^, or Ub^ext^KxR mutants were transformed with pGalUb-R-*LacZ* and analyzed by βgal activity assay. The asterisk (*) denotes statistical significance between Ub^ext^ and EV (p = 0.0239). The cross (+) indicates statistical significance between Ub^ext^ and Ub^ext^K29/48R (p = 0.032). There is no statistically significant difference between Ub^ext^ and Ub^ext^K11R (p = 0.1602). *Lower*: Corresponding βgal protein levels from the lysates used in the βgal activity assay were detected by SDS-PAGE and western blot using an anti-βgal antibody. (C) Ubiquitin conjugation of Ub^ext^ is not necessary for the impaired degradation of the UFD substrate Ub-P-βgal. Cells containing EV, Ub, Ub^ext^, or Ub^ext^KxR mutants were transformed with pGalUb-P-*LacZ* and analyzed by βgal activity assay. The asterisk (*) denotes statistical significance between Ub^ext^ and EV (p = 0.0055). There is no statistically significant difference between Ub^ext^ and Ub^ext^K48/29R (p = 0.4558). (D) Ub^ext^-ubiquitin conjugation is not necessary to impair the degradation of a second UFD substrate, Ub^G76V^-GFP. Cells containing EV, Ub, Ub^ext^, or Ub^ext^KxR mutants were transformed with Ub^G76V^-GFP and analyzed by SDS-PAGE and western blot using an anti-GFP antibody. The blot was reprobed for Pgk1p as a loading control.

UPS-mediated protein degradation is a selective process and polyubiquitination is the signal which targets proteins to the proteasome for degradation [41],[42]. Therefore, we asked whether blocking the ubiquitination of Ub^ext^ would alleviate the associated UPS inhibition. Polyubiquitination can occur on multiple lysine residues of ubiquitin [43]. We mutated four of the lysine residues typically utilized for polyubiquitination by changing them to arginine (referred to as Ub^ext^KxR). Ubiquitin conjugation of Ub^ext^ was visualized by a distinct laddering pattern on a western blot (Figure 2B, black arrows). While none of the single point mutations prevented ubiquitination of Ub^ext^, the double lysine mutant, Ub^ext^K29/48R, did prevent the conjugation (Figure 4A, black arrows).

We evaluated the degradation of the UPS substrates in the presence of the Ub^ext^KxR mutants. The expression of each single Ub^ext^KxR mutant stabilized the N-end rule substrate, R-βgal (Figure 4B). However, the expression of the Ub^ext^K29/48R double mutant allowed for better degradation of the reporter protein, suggesting that the ubiquitination of Ub^ext^ is necessary to impair the degradation of the N-end rule substrate. The steady state levels of βgal protein were detected by western blot and corroborated the result of the βgal activity assay (Figure 4B, lower).

Next, we evaluated the degradation of the UFD substrate in the presence of the Ub^ext^KxR mutants. Each Ub^ext^KxR mutant, including the double mutant (Ub^ext^K29/48R), impaired the degradation of the UFD reporter protein Ub-P-βgal (Figure 4C). Since these data contradict the effects of Ub^ext^K29/48R on N-end rule substrate stability (Figure 4B) and previously published results with UBB+1 [22], we evaluated another UFD substrate, a ubiquitin-GFP fusion (Ub^G76V^-GFP). Western blot analysis revealed that this UFD substrate was also stabilized by Ub^ext^ as well as each Ub^ext^KxR mutant, including the double mutant (Figure 4D). Taken together, these data demonstrate that the conjugation of Ub^ext^ is necessary to cause impaired degradation of an N-end rule substrate, but mono-Ub^ext^ (i.e. Ub^ext^K29/48R) can still impair the degradation of UFD substrates. Based on these data, we suggest that ubiquitin conjugation to N-end rule substrates and UFD substrates is different. The degradation pathways utilized for these two reporters are distinct [3],[31],[32], however they typically report on the same degradation competence of the proteasome, although differences have been cited under certain circumstances [29],[44],[45]. The observed differences here could be explained if different proteins interact with the substrates to perform the ubiquitin conjugation. Perhaps preformed ubiquitin chains are conjugated *en masse* to N-end rule substrates but ubiquitin is added sequentially to UFD substrates. Thus, in the presence of Ub^ext^K29/48R the substrates would be affected differently. Furthermore, this emphasizes that the mode of ubiquitin conjugation, which remains somewhat of a mystery [46], may be an important factor in the differential ability of the cells to cope with one UPS substrate versus another.

### Disruption of the Hydrophobic Patch of Ub^ext^ Modulates Proteasomal Impairment of a UFD Substrate

**Figure 5:**
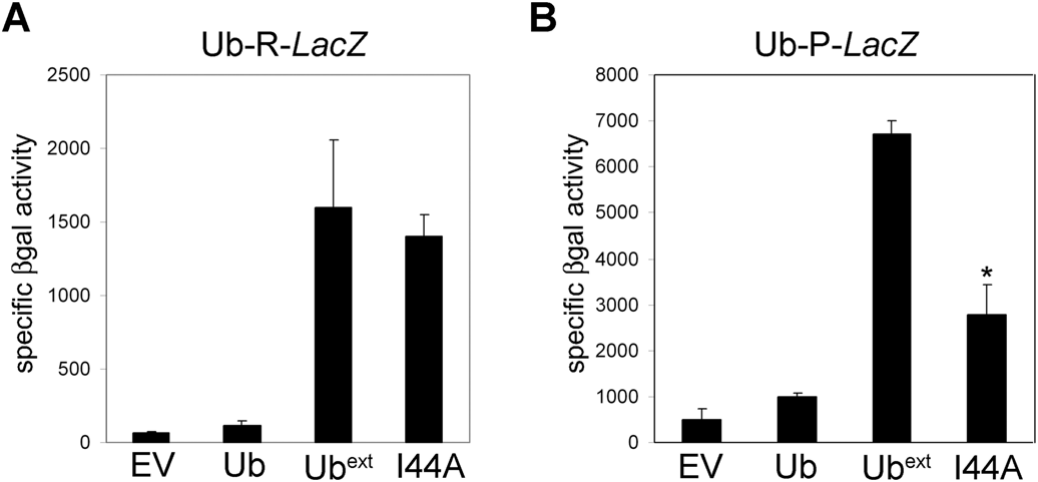
Mutation of the Ub^ext^ hydrophobic patch (I44A) moderately affects proteasomal impairment. (A) Ub^ext^I44A still inhibits N-end rule substrate degradation. Cells containing pGal-Ub-R-*LacZ* were transformed with empty vector (EV), Ub, Ub^ext^ or Ub^ext^I44A (I44A) and the stability of R-βgal was measured by βgal activity assay. (B) Ub^ext^I44A moderately enhances the degradation of a UFD substrate. Cells containing pGal-Ub-P-*LacZ* were transformed with EV, Ub, Ub^ext^ or Ub^ext^I44A (I44A) and the stability of Ub-P-βgal was measured by βgal activity assay. The asterisk (*) indicates statistical significance between Ub^ext^ and Ub^ext^I44A (p = 0.0007).

Our data suggest that Ub^ext^ might be interacting with multiple components of the ubiquitin processing pathway, sequestering proteins required for efficient degradation of proteasome target substrates. Ubiquitin contains a hydrophobic patch (L8, I44 and V70) that is critical for its interaction with many other proteins and the proteasome [47],[48]. The ubiquitin mutation I44A disrupts the hydrophobic patch and this mutant fails to interact with some of its partner proteins [48]. We created a Ub^ext^I44A mutant and tested whether its expression caused UPS impairment. Cells expressing Ub^ext^I44A still stabilized the N-end rule substrate, R-βgal (Figure 5A). However, expression of Ub^ext^I44A resulted in a modest, yet reproducible, increase in the degradation of UFD substrate Ub-P-βgal (Figure 5B). This differential stabilization of the reporters did not occur with different type of mutant ubiquitin, UbΔGG I44A (data not shown). These data suggest that the interaction of Ub^ext^ with other proteins is partially disrupted by mutating the hydrophobic patch and further supports that Ub^ext^ may have multiple interacting partners to impose the UPS impairment.

### Challenging the UPS Decreases Cellular Tolerance to Ub^ext^

**Figure 6:**
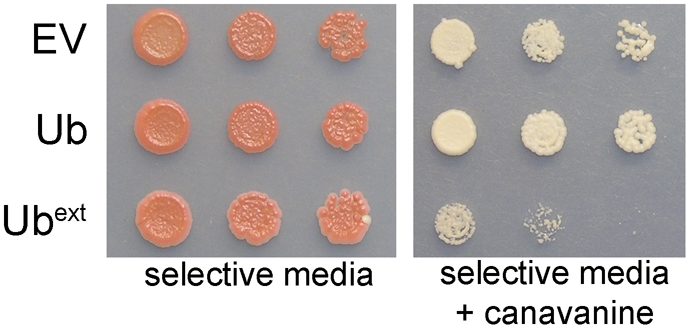
Ub^ext^ expression increases cellular sensitivity to misfolded proteins. Ub^ext^-expressing cells cannot tolerate excess misfolded proteins generated by the incorporation of canavanine. Serial dilutions of cells containing EV, Ub, or Ub^ext^ were spotted onto selective medium and selective medium containing 400 µM canavanine.

The UPS is required for the removal of misfolded proteins. Failure to remove misfolded proteins can lead to aggregation and have detrimental phenotypic consequences. Since the expression of Ub^ext^ exacerbates UPS defects, we next analyzed whether the tolerance to misfolded proteins was decreased in cells expressing Ub^ext^. Canavanine is an arginine analog which becomes incorporated into newly synthesized proteins and causes misfolding [49]. Serial dilutions of cells expressing Ub^ext^ were spotted onto solid medium containing canavanine. Ub^ext^-expressing cells showed impaired growth on canavanine containing medium (Figure 6). This suggests that Ub^ext^ interferes with the ability of the UPS to degrade natural substrates and challenges cell viability when presented with misfolded proteins.

### Expression of Ub^ext^ Affects the Cellular Tolerance to Toxic Aggregates but Does Not Affect Protein Aggregation

**Figure 7:**
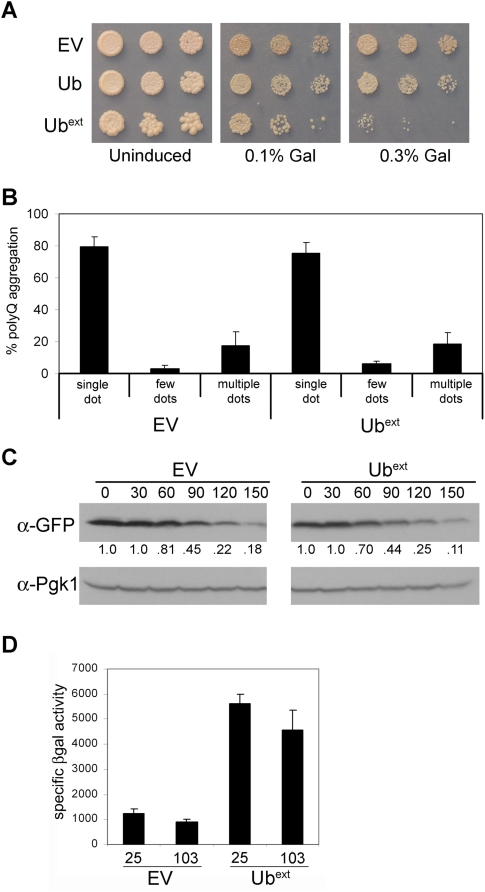
Ub^ext^ expression does enhance the toxicity of polyglutamine expanded protein but does not affect protein aggregation. (A) Cells containing empty vector (EV), Ub, or Ub^ext^ were transformed with a galactose-inducible TOXIC-Q103 construct, which induces cell death in the presence of galactose. Serial dilutions of transformants were spotted onto selective medium (uninduced) and selective media containing either 0.1% or 0.3% galactose. (B) Pre-existing non-toxic HttQ103-GFP aggregates were not altered in the presence of Ub^ext^. Cells transformed with non-toxic HttQ103-GFP and EV or Ub^ext^ were analyzed by fluorescence microscopy. The abundance and pattern of aggregates (dots) was evaluated as described in Materials and Methods using three independent cultures for each sample. Data are expressed as a percentage of the total cells containing aggregates. (C) TOXIC-Q103 protein is not stabilized in the presence of Ub^ext^. Cells expressing TOXIC-Q103 in the absence (EV) or presence of Ub^ext^ were treated with cycloheximide and harvested at the indicated times post translational shut off (in minutes). Cell lysates were analyzed by western blot for the expression of TOXIC-Q103, (which is CFP tagged) using an anti-GFP antibody. Relative protein abundance was quantified as a ratio of the total (below). The membrane was reprobed with an anti-Pgk1 antibody to show protein loading. (D) TOXIC-Q103 protein aggregates do not cause proteasomal impairment. pGal-Ub-P-*LacZ* containing cells with and without Ub^ext^ were transformed with galactose-inducible Q25 or TOXIC-Q103 constructs. The transformants were grown in selective medium containing galactose for 24 hours and the stability of the Ub-P-βgal substrate was measured by βgal activity assay.

We next asked whether misfolded proteins that aggregate would present an additional challenge to cells expressing Ub^ext^. Using tools and properties uniquely available in the yeast system, we sought to determine if Ub^ext^ affects protein aggregation by evaluating both toxic and non-toxic protein aggregates. Since cell death associated with toxic protein aggregates makes it difficult to evaluate the potential contribution of UPS dysfunction, the use of non-toxic aggregates in yeast could provide additional insight as to the direct effects of Ub^ext^. UBB+1 enhanced the aggregation and toxicity of a polyglutamine-expanded protein in cultured mammalian cells [24]. To perform similar experiments in our yeast model, we used a galactose-inducible expanded Huntingtin (Htt) polyglutamine construct, TOXIC-Q103, which creates a toxic protein aggregate [50],[51]. Cells expressing Ub^ext^ could only tolerate a very low amount of TOXIC-Q103, and even with minimal induction, Ub^ext^-expressing cells grew much worse in comparison to control cells (Figure 7A). Interestingly, the expression of Ub^ext^I44A did not result in the same enhanced protein aggregate toxicity (data not shown). Thus, partially alleviating the UPS impairment by altering Ub^ext^ protein interactions relieved the enhanced toxicity.

To determine whether Ub^ext^ expression might affect the aggregates themselves, we imaged a non-toxic version of a polyglutamine-expanded Htt protein fused to GFP (HttQ103-GFP) [52]. Evaluation of these protein aggregates eliminates the complication of cell death associated with toxic aggregates. Previous studies have demonstrated that genetic manipulations, such as altering chaperone levels, can change the abundance and pattern of polyglutamine-GFP aggregates in cells [53]. Thus, we tested whether UPS dysfunction caused by the expression of Ub^ext^ would change the aggregate distribution. Neither the abundance nor the pattern of HttQ103-GFP aggregates was altered in cells expressing Ub^ext^ (Figure 7B). Thus, although the expression of Ub^ext^ did enhance the cellular susceptibility to toxic aggregates, it did not grossly alter the formation or maintenance of non-toxic polyglutamine protein aggregates.

One mechanism by which Ub^ext^ could be enhancing the toxicity of TOXIC-Q103 could involve stabilization of the protein, as the level of expression directly correlates to the amount of toxicity. The stability of TOXIC-Q103 protein was evaluated from cells expressing Ub^ext^ after protein translation was inhibited by cycloheximide. No drastic stabilization of TOXIC-Q103 protein was apparent in cells expressing Ub^ext^ (Figure 7C).

We next asked whether the TOXIC-Q103 aggregates themselves caused UPS impairment. The stability of the UPS reporter protein, Ub-P-βgal, was monitored in cells containing TOXIC-Q103 aggregates in comparison to a non-pathological polyQ25 protein. No stabilization of the reporter was observed in cells harboring the toxic aggregates (Figure 7D). In addition, the UPS impairment caused by Ub^ext^ was not further increased by the presence of TOXIC-Q103 (Figure 7D). Thus, the enhanced toxicity of TOXIC-Q103 caused by Ub^ext^ is not due to additive effects on UPS impairment.

### Enhanced Cellular Toxicity Is Observed with a Second Toxic Protein

To evaluate the generality of the effects of Ub^ext^ on the phenotypic response to toxic protein aggregates, we used a yeast prion protein. Prion proteins in yeast form ordered aggregates that are not harmful to the cells [54]–[56]. Sup35p, an essential translation termination factor, is the protein determinant of the yeast prion [*PSI+*] [55]. The aggregated prion state of Sup35p, [*PSI+*], causes read through of stop codons in translated mRNAs (nonsense suppression). The percentage of read through is low and generally has no deleterious effects to cells grown in rich medium [54]. The presence of the [*PSI+*] prion can be monitored in a strain carrying an *ade1-14* mutant allele with a premature stop codon [57]. In [*psi−*] cells, Sup35p is soluble and functional, and translation is terminated at the premature stop codon in *ade1-14*. Thus, [*psi−*] *ade1-14* cells cannot grow on medium lacking adenine and when grown on rich medium they appear red due to the accumulation of an intermediate in the adenine biosynthetic pathway. Conversely, aggregated Sup35p in [*PSI+*] cells limits the amount of functional Sup35p, thereby causing nonsense suppression of the *ade1-14* premature stop codon and translation of full-length Ade1 protein. These cells are adenine prototrophs and appear white on rich medium. As such, one can evaluate the functional state of Sup35p as it relates to protein aggregation by monitoring the color of the yeast colony. Cells can be maintained stably as [*psi−*], but they can be induced to become [*PSI+*] by over expressing the Sup35 protein.

**Figure 8:**
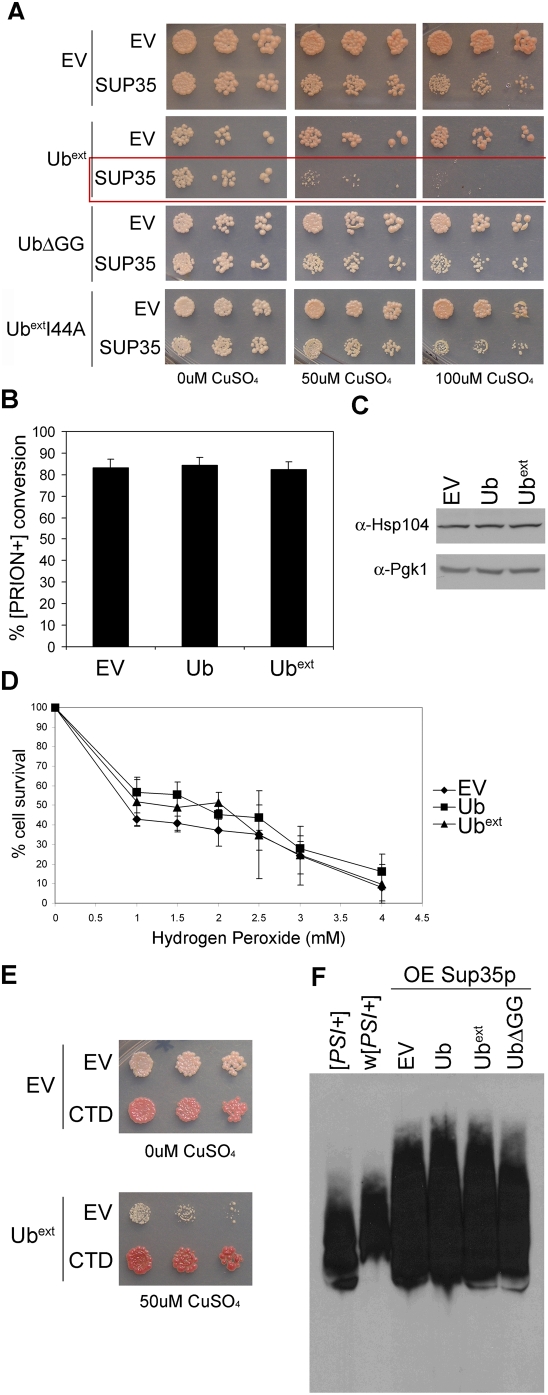
Expression of Ub^ext^ enhances the susceptibility of cells to toxic Sup35p aggregates but does not affect Sup35p aggregation. (A) [*PSI+*] cells expressing Ub^ext^ show reduced cell viability with lower induction of Sup35p. [*PSI+*] cells containing empty vector (EV), Ub^ext^, UbΔGG, or Ub^ext^I44A were transformed with a copper-inducible *SUP35* or EV and analyzed for growth by spotting serial dilutions onto selective media containing 0, 50, or 100 µM CuSO_4_. At 300 µM CuSO_4_, [*PSI+*] cells over expressing Sup35p alone are not viable (not shown). (B) Prion conversion or induction was not enhanced in cells expressing Ub^ext^. [*psi−*] cells expressing pSup35 or the control (EV) were transformed with empty vector (EV), Ub or Ub^ext^ and were analyzed for [*PSI+*] prion formation by monitoring colony color (the appearance of pink colonies). The graph represents the average of three independent cultures in which approximately 2,000 colonies per culture were evaluated for conversion. (C) Hsp104 protein levels are not enhanced in Ub^ext^-expressing cells. Protein lysate from cells containing EV, Ub, or Ub^ext^ were subject to SDS-PAGE and western blot using an anti-Hsp104 antibody. Pgk1p expression was analyzed as a loading control. (D) The expression of Ub^ext^ did not alter cell survival in the presence of oxidative stress. Cells containing EV, Ub, or Ub^ext^ were treated with increasing concentrations of hydrogen peroxide (H_2_O_2_) and the number of viable cells was graphed as a percentage of the untreated. (E) The C-terminal domain of Sup35p (CTD) rescued the enhance susceptibility caused by Ub^ext^ in [*PSI+*] cells over expressing Sup35p. *Upper*: [*PSI+*]-mediated nonsense suppression is alleviated by expression of the CTD. [*PSI+*] cells containing EV show more nonsense suppression (the colony color is light pink). However, [*PSI+*] cells expressing the CTD display efficient translation termination and the colonies are red. *Lower*: [*PSI+*] cells expressing Ub^ext^ in addition to excess Sup35p (induced by 50 µM copper) are rescued from death by the expression of the CTD. (F) Sup35 protein aggregates were not altered by the presence of Ub^ext^. Sup35p aggregates in strong [*PSI+*] ([*PSI+*]) and a weak strain variant of [*PSI+*] (w[*PSI+*]) were analyzed by SDD-AGE. The difference in Sup35p aggregate size of these prion strain variants can be appreciated by this method (compare [*PSI+*] to w[*PSI+*]). Sup35p aggregates from cells expressing excess Sup35p (OE Sup35p) and expressing Ub, Ub^ext^, UbΔGG or containing an EV control were analyzed by SDD-AGE and western blot with an anti-Sup35 antibody.

The [*PSI+*] prion state is not toxic, however, over expression of Sup35p in [*PSI+*] cells inhibits cell growth due to the lack of sufficient translation termination [58]–[60]. As one would expect, the over expression of Sup35p is not toxic to [*psi−*] cells. Thus, the toxicity results from too much aggregation of Sup35p in the prion state. These toxic aggregates provide a means to assess the effects of aggregation of a protein of known function in combination with UPS dysfunction. Since most toxic protein aggregates cause cell death by unknown mechanisms, analyzing the Sup35p aggregates in [*PSI+*] cells provides a unique opportunity to dissect the contributions of the toxic protein aggregates and UPS dysfunction. To evaluate the effects of UPS dysfunction on toxic protein aggregates, [*PSI+*] cells harboring a copper-inducible *SUP35* were transformed with Ub^ext^ and assayed for cell viability (Figure 8A). Ub^ext^-expressing [*PSI+*] cells were more susceptible to the over expression of Sup35p (Figure 8A, *red box*). The expression of Ub^ext^ did not increase basal levels of Sup35p, as determined by SDS-PAGE and western blot analysis (data not shown). Intriguingly, the expression of a different mutant ubiquitin protein, which caused UPS impairment similar to Ub^ext^ (data not shown), UbΔGG, did not enhance the toxicity of Sup35p over expression to the same extent (Figure 8A, compare fourth row to sixth row). These results show that Ub^ext^ enhances the toxicity of protein aggregates by a mechanism that cannot be solely attributed to its effects on UPS impairment, since UbΔGG did not have the same effect. Furthermore, the hydrophobic domain mutant, Ub^ext^I44A, did not result in the same sensitivity to over expressed Sup35p in [*PSI+*] cells (Figure 8A). This suggests that the mechanism by which Ub^ext^ enhances the toxicity of protein aggregates requires interactions with other proteins via the hydrophobic domain.

We evaluated whether the aggregation of Sup35 is altered by the expression of Ub^ext^. A previous study demonstrated that altering ubiquitin levels by either increasing the expression of ubiquitin or preventing its recycling caused an increase in the formation of the [*PSI+*] prion [61]. Furthermore, deletion of a ubiquitin conjugating enzyme also enhanced [*PSI+*] induction [62]. Thus, there is genetic precedence for perturbations of the UPS affecting prion protein aggregation. We asked whether the presence of Ub^ext^ would alter the spontaneous formation of aggregated Sup35p and change cells from [*psi−*] to [*PSI+*]. We did not observe a change in the spontaneous conversion rate (data not shown), which we have measured to be ∼1 in 10^5^ in our strain [63]. We next evaluated the induction of the [*PSI+*] prion state in the presence and absence of Ub^ext^ by over expressing Sup35p in [*psi−*] cells. Since Ub^ext^ perturbs the UPS, one might predict an effect on the induction of protein aggregation. To the contrary, the expression of Ub^ext^ did not enhance the induction of [*PSI+*] (Figure 8B).

### Expression of Ub^ext^ Does Not Cause a Stress Response

The enhanced toxicity of protein aggregates caused by Ub^ext^ could be the result of a general stress response elicited in cells expressing Ub^ext^. The expression of a heat shock element (HSE)-*LacZ* reporter fusion was evaluated in Ub^ext^-expressing cells and no increase in transcription from the HSE promoter at 30°C or at a sub-lethal heat stress of 37°C was observed (data not shown). We next asked whether the presence of Ub^ext^ increased the translation of a stress-inducible heat shock protein. Protein lysate from Ub^ext^-expressing cells and control cells showed similar levels of Hsp104p (Figure 8C), a stress-responsive chaperone. Finally, we tested the tolerance of the cells to oxidative stress. Cells challenged with hydrogen peroxide showed no change in survival in the presence of Ub^ext^ (Figure 8D). These results suggest that Ub^ext^ expression in yeast neither induces a general stress response nor preconditions the yeast to exogenous insult. Therefore, the enhanced susceptibility of Ub^ext^-expressing cells to toxic aggregates is not likely the result of Ub^ext^ inducing a general stress.

### Restoration of Translation Termination Rescues Enhanced Toxicity Caused by Ub^ext^

Overcoming the enhanced protein aggregate toxicity induced by Ub^ext^ expression could shed light on the mechanism by which Ub^ext^ exerts its affects. In attempts to alleviate the Ub^ext^-enhanced aggregate toxicity we conducted a genomic over expression screen using the toxicity caused by over expression of Sup35p in [*PSI+*] cells. We uncovered two rescuing factors, *HSP104* and *SUP45*. Both of these proteins alleviate the toxicity by affecting Sup35p aggregation and the associated phenotypic readout. Over expression of Hsp104p affects the Sup35p aggregates [64] and Sup45p can sequester Sup35p away from the aggregates [65]. To verify that the enhanced protein aggregate toxicity in the presence of Ub^ext^ can be overcome by altering nonsense suppression, we over expressed the C-terminal domain (CTD) of Sup35p, which is sufficient for translation termination but cannot aggregate and form or join the prion state [58],[66]. We found that the expression of the CTD not only restored translation termination of [*PSI+*] cells (Figure 8E, upper), but also alleviated the enhanced toxicity caused by the expression of Ub^ext^ (Figure 8E, lower). These results demonstrate that alleviating the primary deficit in the cells (i.e. the effects of [*PSI+*]) is sufficient to overcome toxicity even in the presence of a modifier (Ub^ext^).

### Model to Explain Cellular Affects of Ub^ext^ on Aggregate Toxicity

We next asked whether Ub^ext^ affected the toxic Sup35p aggregates, since the enhanced cellular toxicity caused by Ub^ext^ and excess Sup35p is [*PSI+*]-dependent. We assayed Sup35p aggregates by semi-denaturing detergent agarose gel electrophoresis (SDD-AGE) [67]. This technique allows large protein aggregates to migrate into the gel and can resolve aggregates of different sizes, as demonstrated by a strain variant of [*PSI+*] (weak [*PSI+*]), which harbors larger Sup35p aggregates than our [*PSI+*] starting strain (Figure 8F). We observed no change in the size of Sup35p aggregates from cells over expressing Sup35p in combination with Ub^ext^ or UbΔGG. One possible explanation for the enhanced toxicity in the presence of Ub^ext^ could relate to a change in the degradation of misfolded Sup35p. As such, we asked whether Ub^ext^ was promoting the accumulation of ubiquitinated-Sup35p. We reprobed the SDD-AGE membrane with an anti-ubiquitin antibody but did not find any ubiquitinated Sup35p by this approach. In additional attempts to look for ubiquitination of Sup35p, we purified Sup35 aggregates [68] but again were unable to detect any ubiquitinated Sup35 protein (data not shown). Other researchers have also noted an inability to identify ubiquitinated-Sup35p [61],[62]. Thus, we conclude that although Ub^ext^ affects the ability of cells to tolerate toxic Sup35p over expression, it is unlikely a direct consequence of blocking the ubiquitination and degradation of Sup35p.

We also evaluated whether the polyglutamine-expanded Htt proteins are ubiquitinated. We were unable to detect ubiquitinated polyglutamine protein in yeast by immunoprecipitation, SDD-AGE or immunofluorescence (data not shown). The inability to find ubiquitinated polyglutamine protein has also been noted previously [52],[69],[70]. Therefore, as with toxic Sup35p, Ub^ext^ is affecting the tolerance to TOXIC-Q103 aggregates by an indirect means.

**Figure 9:**
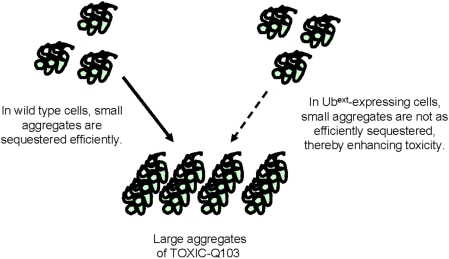
Model for Ub^ext^ affects on toxic protein aggregates. We propose that enhanced protein aggregate toxicity in Ub^ext^-expressing cells is due to the inability of misfolded amyloidogenic proteins to be properly sequestered. The small soluble oligomers are more toxic than the large insoluble protein aggregates. UPS impairment caused by the expression of Ub^ext^ may hinder the rapid sequestration or retention of toxic oligomers into large protein aggregates.

How could Ub^ext^ be affecting the toxicity of protein aggregates if those proteins are not subject to ubiquitination and degradation by the UPS? One possible explanation of the effects of Ub^ext^ on protein aggregate toxicity could be due to a change in the ability to efficiently sequester the toxic proteins into large aggregates (Figure 9). A toxic polyglutamine protein expressed in yeast was rendered non-toxic when sequestered into a single, large aggresome-like structure [70]. Furthermore, a non-toxic polyglutamine protein, which localizes to an aggresome-like structure, became dispersed in ubiquitination-deficient cells. We hypothesize that Ub^ext^ alters the localization of toxic proteins into the large aggregate structures due to its effects on UPS function. The enhanced toxicity could be the consequence of a reduced ability to sequester toxic soluble oligomer species (Figure 9).

### Enhancing Protein Aggregate Toxicity by Increasing the Burden on the UPS

**Figure 10:**
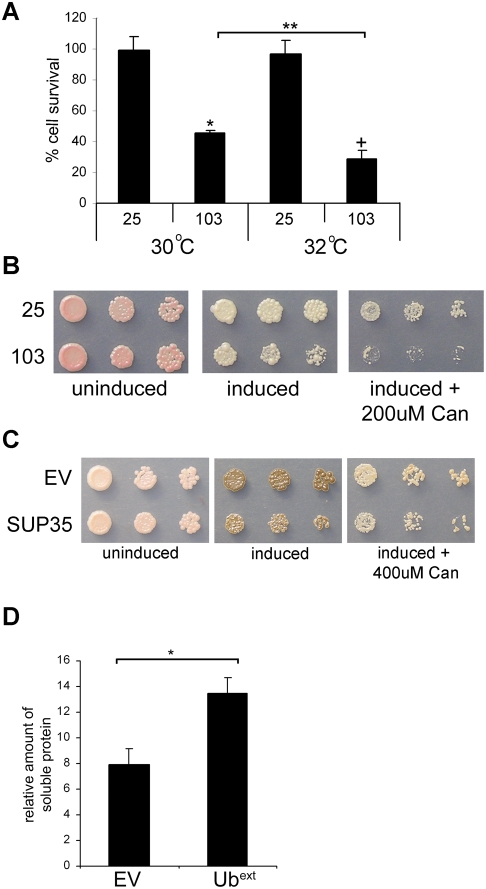
Protein aggregate toxicity is enhanced by perturbations of the UPS and protein aggregate solubility is enhanced by Ub^ext^. (A) Limiting ubiquitination also decreases cellular survival in the presence of TOXIC-Q103. Cellular viability of TOXIC-Q103 (103) or the Q25 control expressed in ts *uba1-204* cells was measured at the permissive temperature (30°C) and a restrictive temperature (32°C). The graph represents the percentage of viable cells from the inducing plates compared to cells grown on non-inducing medium. The asterisk (*) indicates statistical significance between 25 and 103 at 30°C (p = 0.0007), the cross (+) indicates statistical significance between 25 and 103 at 32°C (p = 0.0003), and the double asterisk (**) indicates statistical significance between 103 at 30°C and 32°C (p = 0.0068). (B) Increasing misfolded proteins enhanced toxicity in the presence of TOXIC-Q103. Cell expressing TOXIC-PQ103 (103) and the Q25 control (25) were spotted onto inducing medium and inducing medium containing 200 µM canavanine (Can). (C) The cellular susceptibility of over expressed Sup35p in [*PSI+*] cells in the presence of canavanine is not as detrimental as the co-expression of Ub^ext^. Cells expressing excess Sup35p (induced with 200 µM CuSO_4_) were spotted onto plates containing 400 µM canavanine (Can). Sup35 over expressing cells are slightly less viable in the presence of 400 µM canavanine. All cells died at higher concentrations of CuSO_4_ and canavanine. (D) Cells expressing Ub^ext^ contain more soluble TOXIC-Q103 protein. Cells expressing TOXIC-Q103 in the presence of Ub^ext^ or absence (EV) were lysed and the soluble protein was analyzed by western blot after high speed ultracentrifugation. Densitometry was performed to determine the amount of soluble TOXIC-Q103 protein normalized to the total protein for each sample and graphed in relative arbitrary units. Three independent cultures for each sample were analyzed. The asterisk (*) denotes statistical significance (p = 0.0052).

Based on our hypothesis, we predict that protein aggregate toxicity can be affected by perturbations in ubiquitination or by overwhelming the UPS in general. We took advantage of a temperature-sensitive ubiquitin activating enzyme (E1) mutant (*uba1-204*) [71] to evaluate the effect of an overall reduction in ubiquitination on the phenotypic response to TOXIC-Q103 aggregates. *UBA1* is an essential gene responsible for the first step of the ubiquitination cascade. At the restrictive temperature, the *uba1-204* mutant limits substrate ubiquitination. A recent study demonstrated that polyglutamine protein aggregate patterns were altered in cells expressing the *uba1-204* mutant [70]. *uba1-204* cells expressing TOXIC-Q103 or the control (Q25) were grown in inducing conditions at the permissive (30°C) and restrictive temperatures (32°C) and colony survival was measured (Figure 10A). Cells expressing TOXIC-Q103 showed approximately 50% survival in comparison to those expressing Q25, and this survival was further decreased in conditions of limiting ubiquitination (i.e. 32°C). To directly compare the affect of Ub^ext^ expression on the TOXIC-Q103 aggregates, we measured colony survival as performed above. Cells harboring TOXIC-Q103 aggregates in the presence of Ub^ext^ allowed for only a 7% survival in comparison to TOXIC-Q103 aggregates alone (56% survival). Thus, Ub^ext^ is a more potent modifier of toxic protein aggregates than perturbations in ubiquitination.

Since decreased ubiquitination had an affect on the protein aggregate toxicity, we asked if protein aggregate toxicity could also be enhanced by increasing the burden on the UPS. We measured the viability of cells expressing TOXIC-Q103 or over expressing Sup35p in the presence of canavanine. Serial dilutions of cells expressing Q25 and TOXIC-Q103 were spotted onto inducing media containing canavanine. The effects of the glutamine expansion on cell viability can be seen on inducing plates and in the presence of a UPS burden (canavanine) the toxicity is enhanced (Figure 10B). Over expressed Sup35p in [*PSI+*] cells also shows toxicity and in the presence of canavanine the toxicity is slightly enhanced (Figure 10C). However, canavanine is less potent at enhancing the toxicity of over expressed Sup35p in comparison to the effect of Ub^ext^ (Figure 8A). Nonetheless, perturbations to the UPS in general do appear to enhance protein aggregate toxicity. We propose that this is due to a change in efficient sequestration of toxic proteins into insoluble aggregates (Figure 9).

Since Ub^ext^ enhanced the toxicity of TOXIC-Q103, we tested whether Ub^ext^-containing cells were compromised in their ability to sequester or retain TOXIC-Q103 in the insoluble aggregates. Protein lysates from Ub^ext^ and controls cells (EV) were subjected to high speed ultracentrifugation and analyzed to determine whether Ub^ext^ influences the amount of soluble TOXIC-Q103. Serial dilutions of the total and resulting soluble fraction were applied to PVDF and visualized by western blot. The amount of soluble protein as normalized to total protein was determined by densitometry (Figure 10D). The amount of soluble TOXIC-Q103 was higher in Ub^ext^-expressing cells than wild type cells. Thus, the enhanced toxicity of TOXIC-Q103 in Ub^ext^-expressing cells correlates to an increased pool of soluble protein and supports the model proposed in Figure 9.

### Ub^ext^ Alters Ubiquitination Patterns

**Figure 11:**
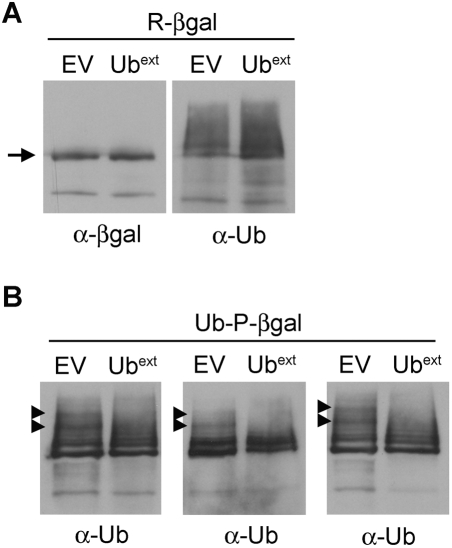
Ub^ext^ alters the ubiquitination pattern of a UPS substrate. (A) R-βgal ubiquitination pattern is not altered in cells expressing Ub^ext^. pGalUb-R-*LacZ* was transformed into proteasome mutant cells (*pre1-1 pre2-2*) expressing Ub^ext^ or EV and R-βgal was analyzed by immunoprecipitation (IP). Membranes were probed with anti-βgal and anti-ubiquitin antibodies. Arrow indicates full length βgal protein. (B) Ub-P-βgal ubiquitination is affected in cells expressing Ub^ext^. Ub-P-βgal IPs were performed as in A. A subtle but reproducible difference in ubiquitination pattern was observed. Three independent IPs are shown. Arrowheads highlight distinct bands present in the EV lanes that are absent in Ub^ext^ lanes.

Since altered ubiquitination affected the distribution of expanded polyglutamine proteins [70] and enhanced the cellular susceptibility to toxic polyglutamine aggregates (Figure 10A), we asked whether Ub^ext^ has a direct effect on the ubiquitination of proteasome substrates. In light of the fact that the toxic protein aggregates are not ubiquitinated, we evaluated the ubiquitination pattern of the UPS reporters. To compare the ubiquitination of these constructs with and without the expression of Ub^ext^, we utilized a temperature-sensitive proteasome mutant strain (*pre1-1 pre2-2*) [28]. This strain is defective in proteolysis and when grown at the restrictive temperature, R-βgal and Ub-P-βgal accumulate (Figure 11A). Striking substrate ubiquitination can be observed in *pre1-1 pre2-2* cells expressing Ub^ext^ and control cells after IP. When we compared the R-βgal substrate ubiquitination in EV and Ub^ext^-containing cells, we did not discern any difference in the ubiquitination pattern (Figure 11A). However, a subtle yet reproducible ubiquitination pattern difference was seen with the Ub-P-βgal substrate (Figure 11B). Three independent IP experiments are shown and two ubiquitinated-βgal bands appear in control cells (EV) which are absent or greatly reduced in Ub^ext^-expressing cells. The altered ubiquitination pattern of some UPS substrates in the presence of Ub^ext^ could change the ability of these proteins to be processed by the proteasome. Furthermore, such changes could be an important modifier of the cellular effects of toxic protein aggregates.

## Discussion

We created a novel model of UBB+1 by constitutively expressing an analogous mutant ubiquitin protein in yeast to investigate the causal relationship between this proteasomal inhibitor and protein aggregation. We demonstrated that the Ub^ext^ mutant was not functional as ubiquitin and was not deleterious to the cells. Importantly, the expression of Ub^ext^ in yeast caused impairment of the UPS. Since proteasome dysfunction can lead to protein aggregation, we were intrigued that the presence of Ub^ext^ served to neither induce nor alter non-toxic protein aggregates in yeast. However, the expression of Ub^ext^ rendered the cells more susceptible to toxic protein aggregates, and this could not be attributed to an increase in general stress elicited by Ub^ext^. We propose that the reduced UPS functionality and altered ubiquitination of UPS substrates in Ub^ext^-expressing cells creates an environment in which toxic amyloidogenic proteins either cannot join or are not maintained as large insoluble aggregates. As a result, protein aggregate toxicity is enhanced due to an increase in soluble or oligomeric toxic protein. Thus, this yeast model system revealed that Ub^ext^ is a phenotypic modifier of toxic protein aggregates. This genetically tractable model provides a platform to further dissect how UBB+1 affects the cellular tolerance to toxic protein aggregates.

The mechanism of UPS impairment caused by UBB+1 is not well understood. We asked whether Ub^ext^ causes a reduction in proteasome activity. Using an unstable ubiquitin-independent substrate (GFP-ODC) [37], we observed no significant change in the activity of the proteasome in Ub^ext^-expressing cells. Based on this result, we suggest that Ub^ext^ is not clogging the core of the proteasome and propose that Ub^ext^ is interacting with other components of the ubiquitin processing cascade or with the regulatory cap of the proteasome. We hypothesized that disrupting the interaction of Ub^ext^ with component(s) of the ubiquitin processing pathway would alleviate the proteasomal impairment. Mutational analysis revealed that ubiquitin conjugation and the hydrophobic patch affect the extent to which Ub^ext^ causes UPS impairment. Interestingly, the effects were distinct with different substrates. This supports the idea that Ub^ext^ is interacting with multiple components of the UPS; reduction of its interaction via the hydrophobic patch or elimination of its ubiquitination weakened some of the observed effects but not others.

Previous studies have investigated the connection between UPS dysfunction and protein aggregation, especially in the context of protein conformational disorders [72]. It remains difficult, however, to discern the precise nature of the causal relationship between protein aggregation and proteasomal impairment. Evidence that UBB+1 and other disease-associated mutations in the UPS can cause proteasomal impairment and increase protein aggregation supports the idea that proteasome dysfunction plays a stimulatory role in protein aggregation. However, in some cases, such as that with mutant Parkin in familial Parkinson's Disease, decreased UPS function is not associated with protein aggregation [8]. Using non-toxic protein aggregates in yeast, we have demonstrated that a UBB+1-like protein, Ub^ext^, neither induced nor changed protein aggregates. Our results provide evidence that a compromised UPS does not necessarily affect protein aggregation *per se* but can cause phenotypic effects by decreasing cellular tolerance to deleterious protein aggregates.

We hypothesize that Ub^ext^ is altering the sequestration of aggregated proteins (Figure 9). Due to the altered substrate ubiquitination and the general UPS impairment caused by Ub^ext^, misfolded proteins are not efficiently degraded and somehow perturb the sequestration of amyloidogenic proteins into the insoluble aggregates which may have a protective function. How the UPS functionality plays a role in the ability of the cell to efficiently sequester non-ubiquitinated proteins remains to be elucidated. One recent study suggests that different cellular compartments retain aggregates of ubiquitinated and non-ubiquitinated proteins and a reduction in UPS activity can cause a change in this localization [69]. If proper localization of aggregated proteins protects the cell from smaller toxic oligomeric species [73],[74], then the inability of toxic oligomers to be efficiently sequestered would be deleterious (Figure 9). Indeed, the expression of Ub^ext^ resulted in an increase in the relative amount of soluble TOXIC-Q103 protein (Figure 10D) and the combination of Ub^ext^ and TOXIC-Q103 was more deleterious to cell survival (Figure 7A). Further evidence to support the idea that the redistribution of aggregates can lead to cell death comes from a recent report investigating the nature of the aggregates formed in response to the expression of expanded polyglutamine protein in yeast [70]. A single large aggregate, an aggresome-like structure, was formed by polyglutamine proteins that were not toxic to the cells. When the large aggregate was unable to form, multiple small aggregates were observed and the appearance of these correlated with toxicity. Thus, the single large aggregate appears to be protective against polyglutamine protein aggregate toxicity. Among the cellular factors found that could disrupt the formation of the single aggregate when mutated were two ubiquitin-associated proteins. Furthermore, limiting general cellular ubiquitination by the *uba1-204* mutant also disrupted the formation of the large aggregate [70]. We show that *uba1-204* enhanced the cellular toxicity of the toxic polyglutamine aggregates used in our study (Figure 10A). Taken together, the data support the proposed model of the effect of Ub^ext^ on protein aggregate toxicity (Figure 9).

Since Ub^ext^ causes UPS impairment and a change in ubiquitination of substrates, this could cause the mis-handling or redistribution of some ubiquitin-conjugated proteins and hinder toxic protein aggregates from being rapidly sequestered, resulting in enhanced cell death (Figure 9). Thus, even though the toxic protein aggregates may not be substrates of the UPS, perturbations in the processing of normal UPS substrates may affect cellular tolerance to toxic aggregates. Our data suggest that all perturbations in the UPS are not equally potent at altering the cellular tolerance to toxic aggregates. Therefore, we conclude that the magnitude of the enhanced protein aggregate toxicity in the presence of the extended mutant ubiquitin is exceptional. This is likely due to its interactions with other proteins and supports further that UBB+1 may be a potent disease modifier.

Since protein conformational disorders result from a combination of cellular perturbations, often including the unknown affects of aging, then eliminating individual modifiers or enhancers may prove useful for disease therapy. Obviously, alleviating the primary causative agent, when known, could prove to be the most beneficial. For example, when we used the Sup35p toxic aggregate model we were able to rescue the Ub^ext^-enhanced toxicity by restoring the loss of function caused by Sup35p sequestration into aggregates. However, in many protein conformational diseases, the function of the proteins found in the aggregates and cellular toxicity is not understood. Therefore, investigating ways to alleviate the effects of known modifiers represents an important therapeutic avenue for disease treatment and prevention. The insight gained by developing a yeast model of UBB+1 has provided a means to further investigate the role of protein aggregate compartmentalization in toxicity, which may underlie some of the effects observed in cells or tissues experiencing chronic UPS impairment. The identification of UBB+1-interacting proteins may allow for the elucidation of the mechanism whereby a natural modifier of UPS function affects cellular tolerance to toxic protein aggregates.

## Materials and Methods

### Strains

Yeast strains were grown and manipulated by standard techniques [75]. Unless otherwise indicated, all yeast strains used in this study were derivatives of 74-D694 (*MATa or MATα ade1-14 trp1-289 his3Δ-200 ura3-52 leu2-3,112*) [64]. The Δ*ubi4* strain was created by PCR amplification of the antibiotic resistance marker KanMX4 with primers A and B and subsequent transformation of the resulting product into 74-D694. For all primer sequences, see Table 1. The *Δubp14* strain was created by PCR amplification of BY4741 *Δubp14* genomic DNA with primers C and D and subsequent transformation of the resulting product into 74-D694. The proteasome mutant strain, WCG4-11/22a (*MATa his3-11,15 leu2-3,112 ura3 pre1-1 pre2-2*) and control strain, WCG4a (*MATa his3-11,15 leu2-3,112 ura3)* were a kind gift of P. Coffino [37]. The 74-D694 [*PSI+*]-inducible prion strain [*psi−*] [*RNQ+*] and the weak [*PSI+*] strain variant were a kind gift from S. Liebman [76]. A 74-D694 [*PSI+*] [*RNQ+*] strain was used in the PQ toxicity study. The *uba1-204* strain was a kind gift from R. Deshaies [71].

**Table 1:** Primer sequences.

Code	Used to make (enzyme)	Sequence (5′ orientation)
A	*ubi4* deletion	GTATTACCCGGCTTCGCGAAAATAGTGAACGTCATAGTATAAGACGATTCATCGATGAATTCGAGCTCG
B	*ubi4* deletion	GGGGTATATATAGAGAGGCTCCGGGTTTTGCCACCTTTGAATTCGCCTGCCGTACGCTGCAGGTCGAC
C	*ubp14* deletion	CACTTGATGAAATCACAGTGAAAAGCG
D	*ubp14* deletion	CGATAGATTTGATCATACACATATAATGC
E	5′ Ub (*Xba*I)	GCT CTA GAA TGC AGA TTT TCG TCA AGA C
F	3′ Ub^ext^ (*Bam*HI)	CGGGATCCTTAACAAAGATCTGCATACCACCTTAGCCTTAGCACAAGATGTAAGG
G	5′ Ub (*Bam*HI)	CGGGATCCATGCAGATTTTCGTCAAGAC
H	3′ Ub (*Sal*I)	GCGTCGACTCAACCACCTCTTAGCCTTAG
I	5′ Ub^ext^ K11R (*Bam*HI)	CCGGATCCATGCAGATTTTCGTCAAGACTTTGACCGGTAGAACCATAACATTGG
J	3′Ub^ext^ (*Sal*I)	CGGTCGACTTAACAAAGATCTGCATACCACCTTAGCCTTAGCACAAGATGTAAGG
K	5′ Ub^ext^ K29R*	GAAGGTATCCCTCCAGATCAAC
L	3′ Ub^ext^ K29R*	CTTGTCTTGAATTcTCGACTTAACGTTGTCGATG
M	5′ Ub^ext^ K48R*	ACGGTAGAACGCTGTCTG
N	3′ Ub^ext^ K48R *	CTTCTAGCTGtcTACCGGCAAAG
O	3′ Ub^ext^ K63R∧	GATGTAAGGTGGACTCCCTCTGAATGTTGTAATC
P	3′ Ub^ext^+GG (*Sal*I)	GGCGGTCGACTTAACCACCACAAAGATCTGCATACCAC
Q	3′ UbΔGG (*Sal*I)	GGCGGTCGACTTATCTTAGCCTTAGCACAAG
R	3′ Ub^ext^I44A∧	CTTACCGGCAAAGGCCAATCTTTGTTG
S	5′ Ub^ext^I44A∧	CAACAAAGATTGGCCTTTGCCGGTAAG

*: Used in three-way ligation with cut p423TEF vector.

**∧:** Used in bridge-PCR and cloned into cut p423TEF vector.

### Plasmids

All plasmids were created using standard molecular biology protocols [77] and verified by DNA sequencing. For primer sequences, refer to Table 1. Where appropriate, the enzyme used is listed parenthetically. To create p413TEFUb^ext^, ubiquitin was PCR amplified from 74-D694 genomic DNA using primers E and F and cloned into p413TEF [78] at *Xba*I and *Bam*HI. To create p413TEFUb, ubiquitin was PCR amplified from 74-D694 genomic DNA using primers G and H and cloned into p413TEF at *Bam*HI and *Sal*I. Ub^ext^ was subcloned from p413TEFUb^ext^ to p423TEF and p426TEF at *Spe*I and *Bam*HI. Ubiquitin was subcloned from p413TEFUb to p423TEF and p426TEF at *Sal*I and *Bam*HI. All Ub^ext^ amino acid substitutions (p423TEFUb^ext^K11R, Ub^ext^K29R, Ub^ext^K48R, Ub^ext^K63R, Ub^ext^K29/48R, Ub^ext^I44A) were created using either three-way ligation or bridge PCR into p423TEF using p423TEFUb^ext^ as a template (except for the p423TEFUb^ext^K29/48R mutant which utilized p423TEFUb^ext^K29R) and following standard molecular biology techniques [77]. p423TEFUb^ext^+GG was created by PCR amplification of ubiquitin DNA with primers G and P and cloned into p423TEF at *Bam*HI and *Sal*I. p423TEFUbΔGG was created by PCR amplification of ubiquitin DNA with primers G and Q and cloned into p423TEF at *Bam*HI and *Sal*I. The 4xHSE-*LacZ* plasmid was a kind gift of S. Lindquist. *In vivo* UPS functionality was measured using Ub-X-*LacZ* reporters: pGal-Ub-M-*LacZ*, pGal-Ub-R-*LacZ*, and pGal-Ub-P-*LacZ* [30]. The ubiquitin-independent proteasome substrates, p416ADH1GFP-mODC and p416ADH1GFP-mODC^C441A^ were a kind gift from P. Coffino [37]. The *UBI4_promoter_-LacZ* reporter was a kind gift from M. Altmann [79]. [*PSI+*] induction assays used the inducer plasmid pEMBL Sup2 (referred to as pSup35 in this manuscript) [58]. Non-toxic polyglutamine aggregation assays used p416GPD polyQ103-GFP [52], referred to as HttQ103-GFP in this manuscript. Toxic polyglutamine aggregation assays employed p416Gal FLAG103Q-CFP (referred to as TOXIC-Q103) and p416Gal FLAG25Q-CFP (referred to as Q25) (kind gift M. Duennwald) [50],[51]. For the toxicity assay in [*PSI+*] cells, Sup35p was over expressed from a copper inducible promoter. pRS315Cup-*SUP35* was generated by cloning Cup1-*SUP35* between *Xho*I and *Sac*I. pRS316-TEF-CtermSup35 contains only the C-terminal domain (amino acids 254–685) of Sup35 and was created by subcloning TEF-CtermSup35 from pRS306TEF-CtermSup35 [80] at *Hind*III and *Sac*I.

### Protein Analyses

Protein lysates were analyzed by standard SDS-PAGE. Protein lysis followed the β-galactosidase assay (see below). The following antibodies were used: Ubiquitin (PD41) (Santa Cruz sc-8017), Hsp104 (kind gift of S. Lindquist), GFP (kind gift of M. Linder), β-galactosidase (Promega Z378A), Pgk1 (Molecular probes A6457), and Sup35 (kind gift of S. Lindquist) [81]. Large Sup35 protein aggregates were separated by SDD-AGE as previously described [82] with modifications previously described [63]. Sup35p over expression was achieved by growing the cultures in 50 µM copper sulfate overnight. Immunoprecipitations were carried out as previously described [83] using 5 µl of mouse anti-β-galactosidase. TOXIC-Q103 protein stabilization was measured after a six hour induction (2% galactose and 1% raffinose containing media) in the presence of 0.5 mg/ml cycloheximide in cultures with equal numbers of cells.

The relative amount of TOXIC-Q103 soluble protein was determined by slot blot. Cells containing TOXIC-Q103 and either EV or Ub^ext^ were grown overnight in selective medium, washed in inducing medium containing 2% galactose/1% raffinose and induced for 14–16 hours. Cells were harvested and lysed with glass beads in PEB (250 mM Tris HCl pH 7.5, 50 mM KCl, 10 mM MgCl2, 1 mM EDTA, 10% glycerol, 10 mM PMSF, 5 µg/ml Aprotinin, Roche Protease cocktail inhibitor (Roche)). Equal protein (100 µg) from EV and Ub^ext^-containing cells was subjected to ultracentrifugation (80,000 rpm for 30 minutes at 4°C). Serial dilutions of the supernatant and total fractions (diluted 1/10) were applied to activated PVDF and probed with an anti-GFP antibody. The supernatant fraction and corresponding total fractions were quantified using Image J software and graphed as normalized arbitrary units.

### β-Galactosidase Assays

UPS functionality was determined by the degradation of Ub-*LacZ* fusions [30] using Galacto-light™ (Applied Biosystems). Cells containing pGal-Ub-M-*LacZ*, pGal-Ub-R-*LacZ* and pGal-Ub-P-*LacZ* were grown in selective medium for 24 hours. The cultures were washed three times in selective medium containing 2% galactose / 1% raffinose and grown overnight in the 2% galactose / 1% raffinose. The cultures were harvested and lysed in Galacto-light Lysis Solution using glass beads. Cell lysate was pre-cleared for 30 seconds at 6,000 rpm at 4°C. In a flat bottom, black-sided 96-well dish, 70 µl of Galacto Reaction Buffer was added to 10 µl of protein lysate and incubated for 60 minutes at room temperature. Luminescence was read immediately after the addition of 100 µl of Light Emission Accelerator. Luminescence values were normalized to protein concentration as determined by Bradford reagent (BioRad). Error bars in all βgal activity assays represent the standard deviation from three independent cultures for each sample. The TOXIC-Q103 protein βgal activity assay was conducted as described above using a TRP1 version of pGal-Ub-P-*LacZ* (subcloned into p424Gal vector) with a 24 hour induction. All statistical analyses were conducted using Student's T-Test.

### Microscopy

Polyglutamine aggregation was monitored by GFP fluorescence in a 74-D694 [*PSI+*] [*RNQ+*] strain background. Three independent samples of mid-log phase cells containing p416GPD polyQ103-GFP [52] and p423TEF EV or p423TEF Ub^ext^ were visualized. Individual fluorescent cells were evaluated for a single aggregate, few aggregates (2–3 per cell) or multiple aggregates (greater than 3 aggregates per cell) as previously described [53]. Approximately 200 cells were analyzed for each sample in triplicate. Error bars represent the standard deviation.

### Phenotypic Analysis

#### Hydrogen peroxide resistance

An equal number of mid-log phase cells containing p423TEF EV, p423TEF Ub or p423TEF Ub^ext^ were treated with various concentrations (1 mM, 1.5 mM, 2 mM, 2.5 mM, 3 mM, and 4 mM) of hydrogen peroxide for 30 minutes at 30°C in liquid selective medium. Cells were diluted (1∶5000) and plated on selective medium. Viable cells were counted and normalized to the non-treated sample. Error bars represent the standard deviation of three independent cultures for each construct in each condition.

#### Proteasome mutant strain synthetic lethality

The proteasome mutant strain (WCG11-22a) and control strain (WCGa) containing p423TEF EV, p423TEF Ub or p423TEF Ub^ext^ were grown overnight in selective medium at 30°C and five-fold serial dilutions of the cultures were spotted on selective medium and grown at 30°C and 37°C.

#### Canavanine treatment

Cells containing p423TEF EV, p423TEF Ub or p423TEF Ub^ext^ were grown overnight in selective medium. Five fold serial dilutions of cultures were spotted onto selective medium containing 200 or 400 µM canavanine. Cells containing TOXIC-Q103, Q25, pRS315EV or pRS315Cup-Sup35 were grown overnight in selective medium. Five fold serial dilutions of cultures were spotted onto selective media containing the indicated amount of copper sulfate and canavanine or galactose and canavanine.

#### [PSI+] induction

Three independent cultures of 74-D694 [*psi−*] [*RNQ+*] cells containing pEMBL Sup2 [58] in addition to p423TEF EV, p423TEF Ub or p423TEF Ub^ext^ were grown overnight in selective medium to an OD_600_∼1.5. The cultures were diluted and plated on YPD, where ∼2,000 colonies were scored for prion induction. Error bars represent the standard deviation.

#### Toxic polyglutamine aggregation

[*PSI+*] [*RNQ+*] cells containing p416Gal FLAG103Q CFP or p416Gal FLAG25Q CFP [50],[51] and p423TEF EV, p423TEF Ub, or p423TEF Ub^ext^ were grown overnight in selective medium. The cultures were diluted five-fold and spotted on selective media containing 0.1% or 0.3% galactose with 1% raffinose.

#### Toxic Sup35p over expression

74-D694 [*PSI+*] [*RNQ+*] cells containing pRS315Cup-EV or pRS315Cup-Sup35 and p423TEF EV, p423TEF Ub^ext^, p423TEF UbΔGG or p423TEF Ub^ext^I44A were grown overnight in selective medium. Cultures were diluted five-fold and spotted on selective medium containing 50 µM or 100 µM copper sulfate. pRS315TEF-CtermSup35 or control pRS315 EV were transformed into Ub^ext^-expressing cells containing pRS315Cup-Sup35 and plated on selective medium containing 50 µM copper sulfate.
